# The Role of m6A Ribonucleic Acid Modification in the Occurrence of Atherosclerosis

**DOI:** 10.3389/fgene.2021.733871

**Published:** 2021-09-16

**Authors:** Jie Fu, Xinghui Cui, Xiaoyun Zhang, Min Cheng, Xiaoxia Li, Zhiliang Guo, Xiaodong Cui

**Affiliations:** ^1^ School of Clinical Medicine, Weifang Medical University, Weifang, China; ^2^ School of Basic Medicine Sciences, Weifang Medical University, Weifang, China; ^3^ Institute of Stem Cell and Regenerative Medicine, Department of Basic Medicine, Qingdao University Medical College, Qingdao, China; ^4^ The 80th Group Army Hospital of Chinese People’ Liberation Army, Weifang, China

**Keywords:** N6methyladenosine (m6A) modification, atherosclerosis, methyltransferase, erasers-demethylases, vascular endothelial cells

## Abstract

The N6-methyladenosine (m6A) modification is the most abundant epitranscriptomic modification in eukaryotic messenger RNA (mRNA). The m6A modification process is jointly regulated by various enzymes and proteins, such as methyltransferases, demethylases and related m6A-binding proteins. The process is dynamic and reversible, and it plays an essential role in mRNA metabolism and various biological activities. Recently, an increasing number of researchers have confirmed that the onset and development of many diseases are closely associated with the molecular biological mechanism of m6A RNA methylation. This study focuses on the relationship between m6A RNA modification and atherosclerosis (AS). It thoroughly summarizes the mechanisms and processes of m6A RNA modification in AS-related cells and the relationships between m6A RNA modification and AS risk factors, and it provides a reference for exploring new targets for the early diagnosis and treatment of AS.

## Introduction

The morbidity and mortality associated with cardiovascular diseases are increasing every year. Atherosclerosis (AS) is the underlying cause of cardiovascular diseases such as myocardial infarction, stroke, and coronary heart disease (Virani et al., 2020). AS refers to the continuous buildup of plaques composed of lipids and fibrous components in the vascular wall and eventually the formation of atherosclerotic necrotic lesions. Additionally, AS is considered a systemic chronic inflammatory disease caused by the activation of inflammatory cells or inflammatory cytokines after cell injury (Fu et al., 2017; Zhu et al., 2018). Unfortunately, however, the mechanism of AS is still unclear.

Recently, epitranscriptomics has become a prominent and emerging research field in explaining AS pathogenesis. An increasing number of studies have shown that epitranscriptomic modification is closely associated with the metabolic processes of tissues and cells in AS (Herrington et al., 2016; Quiles-Jiménez et al., 2020). This article reviews the occurrence of the N6-methyladenosine (m6A) modification in tissues and cells during the development of AS, as well as the relationships between the m6A modification and AS risk factors, such as oxidative stress, obesity, and smoking, to provide new ideas for the study of molecules targeting AS.

## The m6A Ribonucleic Acid Modification

The use of m (Dominissini et al., 2012)A-specific methylated RNA immunoprecipitation and next-generation sequencing (MeRIP-Seq) has significantly increased the efficiency of identifying m6A-modified target mRNAs. Transcriptome-wide sequencing methods are vital for determining methyl modification sites, quantitatively analyzing modifications, and identifying regulatory enzymes or proteins associated with m6A (Dominissini et al., 2012). Three types of enzymes are involved in m6A RNA modification: methyltransferases (MTs) (writers), demethylases (erasers), and m6A-related binding proteins (readers) (Shi et al., 2019; Frye et al., 2018). In various cell lines under different processing environments, the expression levels and localization of m6A-related proteins in cells vary. In addition, their diversity, richness, and varied intracellular localizations make RNA molecules intrinsically heterogeneous, making it quite challenging to study the mechanisms of m6A RNA modification (Marinov et al., 2014; Adivarahan et al., 2018). According to the epitranscriptomic modification database (http://mods.rna.albany.edu/) and relevant studies, there are more than 100 epitranscriptomic modifications, among which the m6A modification is the most abundant in eukaryotic cells (Cantara et al., 2011; Jia et al., 2013; Yue et al., 2015). The m6A modification was first identified in 1974 (Desrosiers et al., 1974) and refers to the methylation of the 6th nitrogen atom of adenine, a nucleotide concentrated in stop codons, the 3′ untranslated region (3′-UTR), and the consensus sequence RRACH (R = A/G, H = A/C/U) in exons. However, subsequently, species-specific distributions of m6A signatures have been found in 5′-UTRs (Linder et al., 2015), around stop codons, and even around start codons in Arabidopsis thaliana (Dominissini et al., 2012; Meyer et al., 2012; Luo et al., 2014; Chen et al., 2015; Cao et al., 2016).

The m6A RNA modification process is dynamic and reversible, and it relies on the regulation of MTs, demethylases, and related specific binding proteins. Studies have shown that the m6A modification is associated with RNA metabolism, including RNA cleavage, degradation, and translation (Camper et al., 1984; Klungland and Dahl, 2014; Zhao et al., 2014). Here, we present a summary of m6A-modification-related enzymes and proteins.

### m6A Writers: Methyltransferases

Methyltransferase complexes, also known as encoders or writers, play essential roles in catalyzing methyl groups (writers). These MTs are multicomponent N6-adenosine MT complexes composed of multiple subunits, including methyltransferase-like 3 (METTL3), METTL14, and other critical regulatory factors, such as Wilms’ tumor 1-associated protein (WTAP) and METTL16 (Bokar et al., 1997; Liu et al., 2014; Ping et al., 2014; Koh et al., 2019). METTL3 is a catalytically active subunit that was first identified based on S-adenosylmethionine (SAM) playing a critical role by providing a methyl group (Liu et al., 2014; Wang et al., 2016; Gao et al., 2021). Studies have shown that METTL14 plays an important role structurally supporting RNA binding by providing an RNA-binding scaffold, which synergistically promotes the binding of the transferase to RNA substrates and enhances the complex’s stability (Koh et al., 2019; Gao et al., 2021; Zheng et al., 2013). According to the latest research, METTL14, an important part of the heterodimeric N6-methyltransferase complex, is a noncatalytic subunit (Wang et al., 2016; Liu et al., 2021). In addition to the METTL3-METTL14 complex, a regulatory subunit, WTAP, is noteworthy. Although WTAP does not have methyl catalytic activity, it can bind to the METTL3-METTL14 complex, and plays an essential role in regulating the localization of the METTL3-METTL14 complex to nuclear foci, thereby affecting the efficiency of methyl modification (Ping et al., 2014; Liu et al., 2014). Moreover, the regulation of the process may strongly depend on METTL14 (Liu et al., 2014).

### m6A Erasers: Demethylases

Demethylases are also known as erasers. Fat mass and obesity-associated protein (FTO) and alkylation repair homolog protein 5 (ALKBH5) are both demethylases that belong to the AlkB family (Zheng et al., 2013; Jia et al., 2011) and can remove m6A methylation in an FeII/α-ketoglutarate (α-KG)- and 2-oxoglutarate (2OG)-dependent manner (Gerken et al., 2007).

The FTO oxidation reaction produces two intermediate products, N6-hydroxymethyladenosine (hm6A) and N6-formyladenosine (f6A), and the final decomposition products are adenine and formaldehyde, which function in an FeII/α-KG- and 2OG-dependent manner (Gerken et al., 2007). Notably, hm6a and f6A are not detected in samples treated with only ALKBH5, and ALKBH5 can directly and efficiently demethylate m6A sites with its DBSH domain combined with the ATP domain of DDX3 (Zheng et al., 2013; Shah et al., 2017; Wei et al., 2018). Therefore, the mechanisms by which FTO and ALKBH5 demethylate are different (Zheng et al., 2013; Gerken et al., 2007; Fu et al., 2013). The deletion of ALKBH5 leads to decreased RNA metabolism (Zheng et al., 2013). Several studies have shown that demethylase dysregulation is closely associated with the occurrence or progression of various diseases, such as AS, metabolic syndrome, brain malformation, growth retardation and even cancer (Dina et al., 2007; Scuteri et al., 2007; Fu et al., 2013; Wang et al., 2020a). These topics may be important research hotspots in the future.

### m6A Readers: m6A-binding Proteins

m6A-binding proteins, also known as readers, are proteins that specifically bind to m6A, and they include YT521-B homology (YTH) domain-containing family (YTHDF) proteins and RNA-binding proteins (RBPs) localized in the nucleus. YT521-B proteins contain a YTH domain and can be divided into 2 subfamilies, YTHDF (YTHDF1-3) and YTHDC (YTHDC1-2) (Wang et al., 2014; Zhou et al., 2021). YTHDF1 is an important m6A-binding protein, and Wang et al. (2015) showed that YTHDF1 is positively correlated with the translation efficiency of its target transcripts and the degree of ribosome occupancy. Zhou et al. (2015) found that under certain stressors, such as heat shock, YTHDF2 can inhibit FTO to facilitate the demethylation of m6A at the 5′-UTR, thus stabilizing m6A methylation at the site; in addition, YTHDF2 can reduce the stability of target transcripts (Wang et al., 2015). YTHDF3 is often synergistic with YTHDF1, thereby enhancing translation efficiency, and it is synergistic with YTHDF2, thereby reducing the stability of m6A-modified transcripts (Wang et al., 2015; Shi et al., 2017). In addition, Lan et al. (Roundtree et al., 2017) found that the YTHDC1 level negatively correlated with the level of m6A methylated mRNA in the nucleus and that YTHDC1 directly or indirectly promoted the export of m6A-modified mRNAs from the nucleus to the cytoplasm through SRSF3 and NXF1, thus promoting translation.

In addition to the YTHDF proteins, the heterogeneous nuclear ribonucleoprotein C (HNRNPC) family and insulin-like growth factor 2 (IGF2) mRNA binding protein 2 (IGF2BP2/IMP2) can also act as m6A methylation readers (Huang et al., 2018). HNRNP plays an important role in promoting the maturation of pre-microRNAs (miRNAs) and regulating the stability of target RNAs by binding to m6A-methylated transcripts (Liu et al., 2015). IGF2BP2 is a type 2 diabetes (T2D)-related gene. An increasing number of studies have shown that IGF2BP2 can bind to m6A modifications. In contrast to the role YTHDF2 plays in m6A modifications, IGF2BP2 can promote the structural stability of m6A-modified mRNAs and thus participate in the occurrence of various diseases (Dai et al., 2011).

**FIGURE 1:**
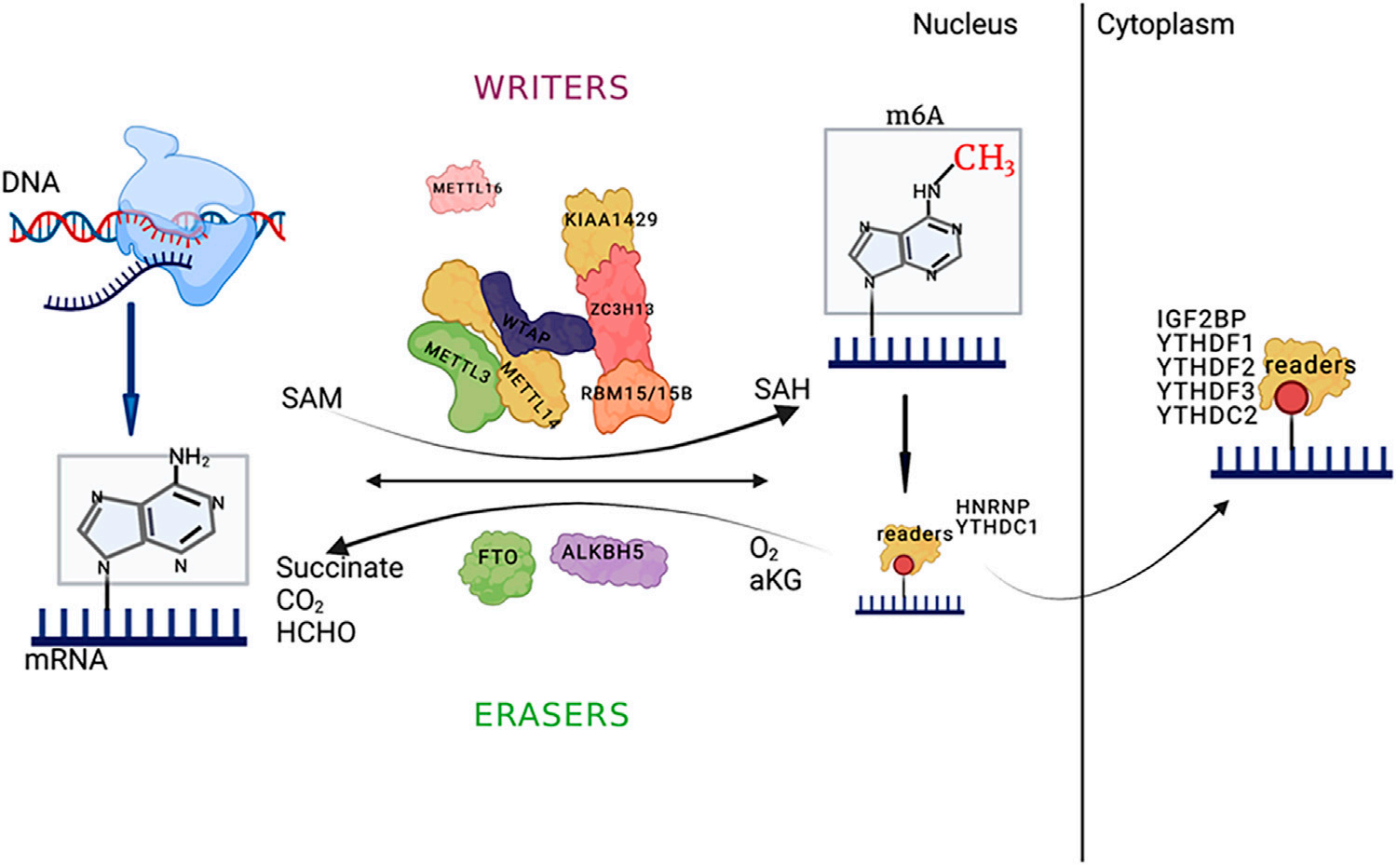
The main processes of m6A RNA modification and the major enzymes involved; and the picture was created with BioRender. The key enzymes that catalyze m6A methylation, such as methyltransferase (MT) complexes (METTL3/METTL14/Wilms tumor 1-associated protein (WTAP) and other regulatory cofactors that form the MT complex), or via methylation by METTL16 alone, are called “writers.” The demethylases that catalyze m6A demethylation (FTO/ALKBH5) are called “erasers.” YTHDF1-3/YTHDC1-2 and IGF2BP1/2/3 are called “readers.” The reaction uses s-adenosylmethionine (SAM) as a methyl donor, and the methyl group is removed to form SAH. The demethylation process for m6A RNA depends on Fe (II)/α-ketoglutarate (α-KG) and 2-oxoglutarate (2OG). The main functions of the binding proteins are to affect splicing, export and translation efficiency. The schematic diagram was created with BioRender.

m6A modification can induce the exposure of RNA-protein binding sites and affect the biological regulation of RNA-protein interactions, referred to as the m6A switch mechanism (Liu et al., 2015). An abundant RNA-binding protein, HNRNPC readily binds to uracil (U) on single-stranded RNA and participates in precursor RNA processing. The m6A modification of single-stranded RNA can induce the exposure of RNA-protein binding sites, thereby promoting HNRNPC binding, affecting substrate mRNA content, and influencing mRNA alternative splicing. These findings provide new directions for epitranscriptomic research (Figure 1).

## Atherosclerosis and the m6A Ribonucleic Acid Modification

AS could cause vascular endothelial cell function or structure change, causes the lining the lower blood lipid and inflammatory cells abnormal deposition, migration of smooth muscle cells lining to the proliferation of macrophage origin and smooth muscle cells source sex foam cells, resulting in the formation of atherosclerotic plaque (Falk, 2006). Chemical modifications provide additional novel functional features for RNA, and one of the most common and characteristic mRNA modifications is m6A. This modification has been shown to be important in many aspects of the mRNA life cycle, from splicing to nuclear transport and stabilization to translational regulation. The relationship between atherosclerosis and m6A modification is highlighted below.

An increasing number of studies have shown that m6A RNA modification can affect the development and progression of AS (Jian et al., 2020; Quiles-Jiménez et al., 2020; Chen et al., 2021). An AS study found that the expression levels of zinc finger NFX1-type containing 1 (ZNFX1) antisense RNA1 (ZFAS1) and the downstream ADAM10/RAB22A were important in the development of vascular inflammation and cholesterol metabolism (Tang et al., 2020). Chen et al. (2016) showed that the m6A modification of ZFAS1 in AS patients was significantly higher than that in a control group and that m6A modification of ZFAS1 was regulated by METTl14. METTl14 affects the expression of downstream ADAM10/RAB22A by affecting the m6A modification of ZFAS1, thus participating in cholesterol metabolism and vascular inflammation and ultimately regulating the occurrence and development of AS (Quiles-Jiménez et al., 2020; Gong et al., 2021). These results provide an important reference for the study of m6A RNA modification and AS.

The occurrence of AS is closely related to the cells associated with the occurrence of plaques, such as vascular endothelial cells, macrophages and SMCs. m6A RNA modification may be involved in AS progression by affecting the functions of these cells. Thus, we have summarized the relationship between the regulation of m6A RNA modification-related enzymes and the cells involved in AS, as shown in Table 1.

**TABLE 1:** Regulation of m6A methylation-related enzymes in cells involved in AS.

Atherosclerosis-associated cells	m6A regulators	Related genes	Effects of m6A regulators	References
Endothelial cells (ECs)	METTL14	miR-19a/PremiR-19a	Proliferation and migration of vascular endothelial cells	Zhang et al. (2020)
	METTL14	FOXO	Inflammatory response of endothelial cells	Jian et al. (2020)
	WTAP/IGF2BP1/IGF2BP3	DSP	Angiogenesis of endothelial cells	Wang et al. (2020b)
	FTO/YTHDF2	FAK	Proliferation, migration and angiogenesis of endothelial cells	Shan et al. (2020)
Macrophages (Mø)	METTL3	has_circ_0,029,589/STAT1	Apoptosis of macrophages/affects macrophage polarization	Guo et al. (2020)
				Liu et al. (2019)
	FTO	NF-κB/STAT1/STAT6/PPAR-γ	Affects macrophage activation	Gu et al. (2020)
	YTHDF2	P38/ERK/NF-κB/STAT1/PPAR-γ/MAP2K4/MAP4K4	Affects macrophage activation/induction of the inflammatory response	Yu et al. (2019)
Smooth muscle cells (SMCs)	METTL3	α-SMA/SM22α/Calponin/SM-MHC/VEGF/HGF/TGF-β/GM-GSF/bFGF/SDF-1	Smooth muscle cell differentiation	Lin et al. (2020)
	WTAP	P16	Inhibition of cell viability, proliferation and migration potential of VSMCs	Zhu et al. (2020)

### m6A Ribonucleic Acid Modification and Vascular Endothelial Cells

AS is often accompanied by structural or functional damage to endothelial cells, so the involvement of vascular endothelial cells in angiogenesis is critical in atherosclerotic vascular repair. Jian et al. (2020) screened and identified m6A-modified mRNA via methylated RNA immunoprecipitation (RIP) sequencing and selected the forkhead box, class O (FOXO) gene as a potential target. Subsequently, the m6A modification of FOXO was found to increase the inflammatory response. However, when METTL14 was knocked out, the expression of inflammation-induced FOXO was significantly reduced. A study by Jian et al. (2020) demonstrated that m6A modification was involved in the induction of inflammatory responses in endothelial cells and played an essential role in the formation of plaques in AS. In addition, WTAP acts as a crucial regulatory subunit in the MT complex, and several studies have shown that WTAP expression affects m6A modification at the mRNA level in endothelial cells. For instance, Wang et al. (2020b) used RNA transcriptome sequencing (RNA-Seq) and found that in brain arteriovenous malformations (AMVs), the expression level of WTAP was significantly decreased but could inhibit endothelial cell angiogenesis. Interestingly, there were no significant differences in the expression of METTL3 or METTL14. MeRIP-Seq was further used to conclude that WTAP affects desmoplakin (DSP) methylation to influence angiogenesis, and the m6A readers IGF2BP1 and IGF2BP3 can stabilize methylated DSP mRNAs and prevent degradation (Wang et al., 2020b). Other studies have shown that the demethylase FTO is significantly increased, and in neovascularized corneas and ECs, the expression of FTO is increased (Shan et al., 2020). The m6A modification of FAK increased under pathological conditions. Interestingly, YTHDF2, an m6A RNA reader, can reduce the stability and increase the decay of m6A-modified RNA (Shan et al., 2020). The above results show that the m6A modification can affect biological functions, thereby causing corresponding diseases.

### m6A Ribonucleic Acid Modification and Macrophages

AS is a chronic inflammatory disease. Macrophages, vital effector cells of the immune system, are inseparable from the vascular inflammatory process in AS (Koelwyn et al., 2018). Circular RNAs (circRNAs) are a new group of noncoding RNAs that always form circular structures with 5′-3′-phosphodiester bonds and do not contain free 5′ or 3′ ends. They are highly stable in cells (Li et al., 2018). A growing body of research suggests that circRNA is closely associated with cardiovascular disease (Kishore et al., 2020; Cao et al., 2020). Studies have shown that the expression of macrophage hsa_circ_0,029,589 in acute coronary syndrome is significantly reduced and that the expression and methylation of METTL3 (an m6A MT) are significantly increased; when METTL3 expression is inhibited, the methylation of hsa_circ_0,029,589 decreases, and the expression of hsa_circ_0,029,589 increases. Interestingly, interferon regulatory factor-1 (IRF-1) overexpression promotes METTL3 expression in macrophages and the m6A methylation of hsa_circ_0,029,589. Therefore, IRF-1 may inhibit the expression of hsa_circ_0,029,589 through hsa_circ_0,029,589 methylation, thereby regulating macrophage apoptosis and controlling the occurrence of inflammation (Guo et al., 2020). In addition, Liu et al. (2019) found that the expression of the m6A MT METTL3 was closely associated with the M1 polarization of mouse macrophages. METTL3 can increase m6A modifications in the coding sequence (CDS) and 3′-UTR of signal transducers and activators of transcription 1 (STAT1), increase the stability of STAT1, and participate in the inflammatory response.

An association between the demethylase FTO and macrophage polarization has also been reported. Gu et al. (2020) found that FTO can affect the nuclear factor kappa B (NF-κB) signaling pathway and that YTHDF2 can affect the stability of STAT1 and peroxisome proliferator-activated receptor-γ (PPAR-γ). When the m6A-binding protein YTHDF2 was knocked out, the mRNA stability and mRNA expression levels of STAT1 and PPAR-γ increased, and both STAT1 and PPAR-γ participated in macrophage activation. In addition, when YTHDF2 was knocked out in lipopolysaccharide (LPS)-stimulated RAW 264.7 cells, the stability of m6A-modified mitogen-activated protein 2 kinase 4 (MAP2K4) and mitogen-activated mitogen-activated protein 4 kinase 4 (MAP4K4) was enhanced. The expression levels of related inflammatory pathways, such as the p38, extracellular signal-regulated kinase (ERK) and NF-κB pathways. And the downstream related inflammatory molecules, such as interleukin (IL)-6, tumor necrosis factor (TNF)-α, IL-1β and IL-12, were increased, suggesting that YTHDF2 regulates the LPS-induced inflammatory process in macrophages (Yu et al., 2019). Similarly, studies have reported that exposure to macrophage mono-(2-ethylhexyl) phthalate (MEHP) can affect the m6A modification of the scavenger receptor B type 1 (SR-B1) gene associated with cholesterol efflux and the m6A modification of related miRNAs and can participate in macrophage-mediated cholesterol efflux (Park et al., 2020), providing strong evidence supporting the involvement of the RNA m6A modification in macrophages in the occurrence of AS.

### The m6A Ribonucleic Acid Modification and Vascular Smooth Muscle Cells

Mature VSMCs have a high degree of plasticity and can transform from a quiescent contractile phenotype to a migratory proliferative and synthetic secretory phenotype. Under the continuous action of a specific environment or stimulating factors, the ability of SMCs to mechanically contract significantly decreases, and the ability to secrete extracellular matrix is enhanced, eventually leading to a thickening of the vascular wall and a narrowing of the lumen (Miano et al., 2021). Studies have shown that hypoxia can affect METTL3 expression and further affect the m6A modification of related factors, such as vascular endothelial growth factor (VEGF) and transforming growth factor (TGF)-beta, thereby inducing the differentiation of adipose-derived stem cells (ADSCs) into VSMCs (Lin et al., 2020). The occurrence of AS and restenosis after angioplasty are closely related to this process (Frismantiene et al., 2018). In a rat model of carotid balloon injury, Zhu et al. (2020) measured m6A modification in SMCs. The results showed that m6A modification in rats with carotid balloon injury was 0.59 times that in rats in the control group and that m6A methylation was reduced. Additionally, quantitative polymerase chain reaction (qPCR) analysis showed that WTAP expression was significantly decreased, and after treatment with total Panax notoginseng saponin (TPNS), WTAP expression increased and m6A modification of the downstream target gene P16 increased, indicating that the WTAP-p16 signaling axis plays a key role in the regulation of endometrial hyperplasia through m6A modification. The study by Zhu et al. (2020) not only examined the effect of TPNS in the treatment of endometrial hyperplasia but also provided potential biomarkers and new research ideas for the treatment of AS.

### Relationship Between m6A Ribonucleic Acid Modification and Atherosclerosis Risk Factors

**FIGURE 2:**
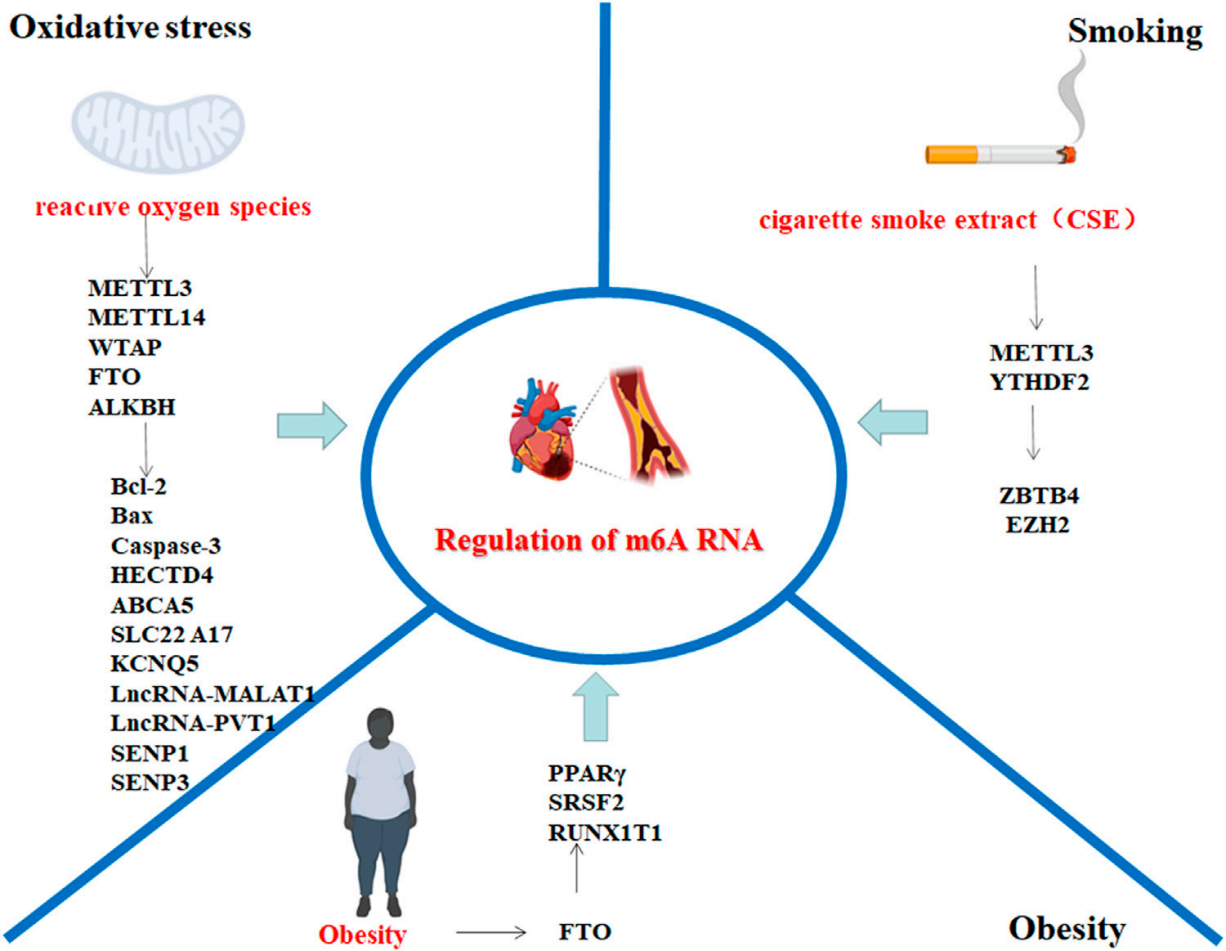
AS risk factors involved in the regulation of m6A methylation-related target genes; the picture was created with BioRender. The genes associated with oxidative stress and m6A RNA modification include HECTD4, ABCA5, SLC22A17, KCNQ5, lncRNA-MALAT1, and lncRNA-PVT1; genes associated with obesity and m6A RNA modification include SRSF2 and PPAR-γ; and genes associated with smoking and m6A modification include ZBTB4, EZH2 and H3K27me3. The above genes may be involved in m6A RNA modification, which may be involved in the AS disease process. The schematic diagram was created with BioRender.

In recent years, substantial progress has been made in the treatment of cardiovascular diseases, but cardiovascular diseases are still the leading cause of death (Iyen et al., 2019; Libby et al., 2013). An increasing number of studies indicate that dynamic m6A modification plays an important regulatory role in cardiovascular diseases, especially AS (Quiles-Jiménez et al., 2020). Strong risk factors for AS, such as oxidative stress, obesity, and smoking, are closely associated with the dynamic regulation of m6A RNA methylation. Research progress on the relationship between m6A RNA modification and AS risk factors is summarized (Figure 2).

### m6A Ribonucleic Acid Modification and Oxidative Stress

Oxidative DNA damage caused by reactive oxygen species (ROS) can be involved in the occurrence of many diseases, and increasing evidence shows that excessive oxidative stress plays a key role in inducing cell damage and promoting AS progression, which is an important cause of cardiovascular diseases (Incalza et al., 2018; Krylatov et al., 2018). Epitranscriptomics plays a critical role in the study of cardiovascular disease. Some studies have shown that the m6A-modified form of the 5′-UTR can affect mRNA translation in a cap-independent manner (Linder et al., 2015; Jackson et al., 2010). These results provide a reliable theoretical basis for studying the relationship between m6A RNA modifications and some pathogenic factors. An increasing number of studies have found that translation in a cap-independent manner is associated with cell stress, infection, cancer and other diseases (Stoneley and Willis, 2004). It has not been reported whether ROS are involved in the process of AS by affecting m6A modification of mRNA and then regulating RNA translation. Wu et al. (Stoneley and Willis, 2004) showed that compared with normal mice, aflatoxin-treated mice had increased ROS levels, which in turn led to the increased expression of the MT METTL3 and the decreased expression of the demethylase FTO in the liver tissue of mice treated with ROS; therefore, the m6A modification of related RNA, such as B-cell lymphoma 2 (Bcl-2), cysteine aspartyl-specific protease (caspase)-3, and Bcl-2-associated X protein (Bax), increased, thus participating in the pathological process of liver injury. Studies have shown that resveratrol may reverse ROS-induced liver injury caused by aflatoxin by reducing RNA methylation (Wu et al., 2020; Ko et al., 2017). Zhao et al. (2019) confirmed that arsenous-acid-induced oxidative stress can affect the expression levels of m6A MT and demethylases, especially the expression levels of WTAP and METTL14, thereby regulating the m6A modification of genetic polymorphisms of C12orf51 (HECTD4), ATP-binding cassette transporter A5 (ABCA5), solute carrier family 22 member 17 (SLC22 A17) and potassium voltage-gated channel subfamily Q member 5 (KCNQ5) genes and influencing the transcription and translation of the genes, thus participating in subsequent pathophysiological processes. Exposure to arsenic is closely associated with the induction of human diseases, such as various skin diseases and cancer (Martinez et al., 2011; Argos, 2015). Cui et al. (Argos, 2015) discovered that the FTO expression level is increased in keratinocytes treated with arsenic and that the m6A RNA modification level decreases. The authors also showed that arsenic can inhibit FTO degradation by inhibiting P62 transcription, consequently promoting FTO stabilization. The changes in epitranscriptomic modification caused by arsenic-induced oxidative damage have great significance in understanding disease progression (Cui et al., 2021).

The RNA demethylase ALKBH5 is also a key regulator in protecting cells from DNA damage and apoptosis during ROS-induced stress. ROS can increase the overall m6A modification of mRNA in human HEK293T and HeLa cells. In one study, ROS inhibited the activity of ALKBH5 via ERK/c-Jun NH 2-terminal kinase (JNK), which promoted the phosphorylation of ALKBH5 on serine residues S87 and S325. Furthermore, phosphorylated ALKBH5 further promotes the interaction between ALKBH5 and small ubiquitin-related modifier (SUMO)ylated E2 UBC9. At the same time, the ERK/JNK/ALKBH5 PTM/m6A axis was activated in hematopoietic stem/progenitor cells (HSPCs), which also contributed to maintaining stable genomic integrity and thereby participated in the DNA damage response (Yu et al., 2021). In addition, in other studies, ALKBH5 is closely associated with sentrin/SUMO-specific proteases 1 and 3 (SENP1 and SENP3) under oxidative stress (Xu et al., 2008; Huang et al., 2009; Wang et al., 2012). In addition, in the presence of cadmium sulfate-induced oxidative damage, FTO expression decreases, the m6A modification of the long noncoding RNAs (lncRNAs) lncRNA-MALAT1 and lncRNA-PVT1 increases, and the expression levels of lncRNA-MALAT1 and lncRNA-PVT1 decrease, which in turn induces biological activities, such as ROS accumulation, malondialdehyde (MDA) accumulation and decreased superoxide dismutase (SOD) activity (Qu et al., 2021). Chromium (Cr) (VI) is a toxic substance, and Lv et al. (2021) found that Cr(VI) can cause mitochondrial damage in spermatogonial stem cells/progenitors, resulting in increased ROS production, autophagy, and loss of cell viability. In turn, this damage may cause various reproductive diseases, such as disorders of spermatogenesis. The authors explored the mechanism and found that the m6A RNA modification level decreased in spermatogonial stem cells/progenitors treated with Cr (VI), while melatonin restored the expression of METTL3. Cr(VI)-induced damage was reversed by restoring METTL3-mediated RNA m6A modification and activating mitofusin 2 (MFN2) and optic atrophy 1 (OPA1), as well as inhibiting the Bcl-2/adenovirus E1B 19-kDa-interacting protein 3 (BNIP3)/NIX mitophagy receptor pathway (Lv et al., 2021). However, studies on the relationship between AS, epitranscriptomics, and ROS are still lacking; their relationship requires further investigation.

### m6A Ribonucleic Acid Modification and Obesity

Poor dietary habits can increase the incidence of nutritional imbalance or obesity and can also seriously threaten cardiovascular and cerebrovascular health. According to statistics from the World Health Organization (WHO), by 2015, there were more than 700 million people with obesity worldwide, indicating that many people are overweight (Morabia and Abel, 2006). FTO was first identified as a demethylase in 2011 and is involved in m6A RNA methylation. Zhang et al. (2015) proposed that FTO was necessary for preadipocyte differentiation and may affect adipocyte differentiation by activating the WNT signaling pathway to inhibit the activity of PPAR-γ. Subsequent studies have shown that FTO gene mutations, such as single nucleotide polymorphisms (SNPs), are closely associated with the probability of obesity in children and adults (Dina et al., 2007; Keller et al., 2017)). In addition, Zhao et al. (2014) found that the m6A-modified region spatially overlapped with mRNA splicing regulatory serine/arginine-rich (SR) protein exonic splicing enhancer binding regions and the serine/arginine-rich splicing factor 2 (SRSF2) mRNA binding clusters, indicating that FTO plays an important role in regulating SRSF2 mRNA splicing and adipocyte differentiation. In addition, FTO has been shown to regulate the splicing of the exon region of adipogenic regulator runt-related transcription factor 1 translocation partner 1 (RUNX1T1) by regulating m6A modification, which provides new insights for the study of m6A modification in adipocyte differentiation (Zhao et al., 2014). Church et al. (2010) found that after FTO overexpression, the fat content and body weight of mice fed a normal diet and a high-fat diet showed dose-dependent increases, confirming that FTO is an important factor involved in the development of obesity. Therefore, in-depth studies of the relationship between obesity and m6A modification are playing key roles in revealing the mechanism of AS, especially intervention targets.

### m6A Ribonucleic Acid Modification and Smoking

Smoking is a risk factor for the development of AS. Increasing evidence shows that cigarette smoke extract (CSE) is not only potentially associated with vascular endothelial dysfunction (Pepine et al., 1998) but can also induce endothelial cell pyrolysis (Wang et al., 2019). In addition, CSE is closely associated with a variety of biological activities, such as LDL oxidation, VSMC proliferation, and inflammatory cell aggregation (Yamaguchi et al., 2005; Guo et al., 2017; Zong et al., 2019). In recent years, there have been several studies on the role of CSE in the regulation of dynamic m6A RNA modification in disease mechanisms (Kupsco et al., 2020). Cheng et al. (2021) found that in CSE-treated human bronchial epithelial (HBE) cells, the expression of the MT METTL3 increased, resulting in increased m6A modification of zinc finger and BTB domain containing 4 (ZBTB4) and that m6A-modified ZBTB4 was recognized by YTHDF2, resulting in decreased stability and decreased ZBTB4 expression; interestingly, low ZBTB4 expression can lead to the increased expression of enhancer of zeste homolog 2 (EZH2), which is involved in the induction of the epithelial-mesenchymal transition (EMT). Increasing evidence shows that smoking plays an important role in the regulation of m6A modification, providing a reliable reference for the study and interpretation of the occurrence and development of smoking-induced diseases.

## Conclusion and Prospects

AS is by far the most common vascular disease and is the main cause of coronary heart disease, cerebral infarction, and peripheral vascular disease (Falk, 2006). The development of AS involves interactions among multiple factors, such as oxidative stress and proinflammatory cytokine stimulation (Libby, 2000; Zaman et al., 2000; Singh and Bedi, 2013). Therefore, the mechanism of AS is very complex and is still not fully understood. It is undeniable that cells involved in the development of AS, such as vascular endothelial cells, macrophages and SMCs, play important roles. By regulating the levels of MTs and demethylases, m6A RNA modification can be dynamically affected, and then, through binding to specific m6A-binding proteins, the splicing, folding, transport, translocation, degradation and translation of RNA are affected, ultimately determining the progression and outcome of AS.

This study investigated the relationships between m6A RNA modification and strong risk factors for AS, such as oxidative stress, obesity, and smoking, and found that these factors can affect the m6A modification of specific genes to regulate gene expression and ultimately participate in the occurrence and development of a variety of diseases. Interestingly, the m6A RNA modification is preferentially found at the 5′-UTRs of newly transcribed mRNAs, and the m6A modification level is increased under certain stressful situations. Zhou et al. (2015) found that under heat shock, proteins modified by m6A, such as Hsp70, and the m6A modification level of Hsp70 mRNA in the 5′-UTR were increased, which led YTHDF2 to localize to the nucleus and suppressed FTO expression. Then, they showed that the 5′-UTR, which was m6A modified, could enable translation initiation, and this progress was independent of the m7G cap at the 5′ end (Zhou et al., 2015). Can atherosclerotic risk factors also be considered a stressor that regulates the modification of m6A mRNA and participates in mRNA translation in a cap-independent manner? The stress pathways that regulate m6A modification at the 5′-UTR to influence AS remain unknown. The studies in this field are still insufficient, and extensive study is urgently needed.

In summary, m6A RNA modification is dynamic and reversible and has great importance in the prevention and treatment of disease progression. Further studies are needed to identify additional regulators, in addition to the currently known methylases, demethylases, and related specific m6A-binding proteins, to further explain their relationships with the mechanisms of disease occurrence and development and provide a new direction for research on and the treatment of AS.

## Glossary

AS: atherosclerosis
m6A: N6-methyladenosine
SAM: S-adenosyl-l-methionine
SAH: S-Adenosyl-l-homocysteine
METTL3: methyltransferase like protein 3
METTL14: methyltransferase like protein 14
FTO: fat mass and obesity-associated protein
ALKBH5: alkylation repair homolog protein 5
hm6A: N6-hydroxymethyladenosine
f6A: N6-formyladenosine
YTH: YT521-B homology
YTHDF: YT521-B homology domain-containing family
HNRNPC: heterogeneous nuclear ribonucleoprotein C
IGF2: insulin-like growth factor 2
T2D: type 2 diabetes
U: uracil
ZNFX1: zinc finger NFX1-type containing 1
ZFAS1: zinc finger antisense RNA1
SMCs: smooth muscle cells
RIP: RNA immunoprecipitation
FOXO: fork head box class O
IRF-1: interferon regulatory factor-1
STAT1: signal transducers and activators of transcription 1
PPAR-γ: peroxisome proliferator-activated receptor-γ
LPS: lipopolysaccharide
MAP2K4: mitogen-activated protein kinase kinase 4
ERK: extracellular signal‐regulated kinase
TNF: tumor necrosis factor
IL: interleukin
MEHP: macrophage mono-(2-ethylhexyl) phthalate
SR-B1: scavenger receptor B type 1
VSMCs: vascular smooth muscle cells
VEGF: vascular endothelial growth factor
TGF: transforming growth factor
ADSCs: adipose-derived stem cells
qPCR: quantitative polymerase chain reaction
TPNS: total Panax notoinseng saponin
ROS: reactive oxygen species
Bcl-2: B-cell lymphoma 2
caspase: cysteine aspartyl-specific protease
bax,Bcl-2-associated X protein
ABCA5: ATP-binding cassette transporter A5
SLC22 A17: solute carrier family 22 member 17
KCNQ5: potassium voltage-gated channel subfamily Q member 5
lncRNA: long noncoding RNA
MDA: malondialdehyde
SOD: superoxide dismutase
WHO: world health organization
SNPs: single nucleotide polymorphisms
SR: serine/arginine-rich
SRSF2: serine/arginine-rich splicing factor 2
RUNX1T1: runt-related transcription factor 1 translocation partner 1
CSE: cigarette smoke extract
LDL: low-density lipoprotein
HBE: human bronchial epithelial
EZH2: enhancer of zeste homolog 2
EMT: epithelial-mesenchymal transformation
H3K27me3: histone 3 lysine 27 trimethylation
DSP: desmoplakin
IGF2BP2: insulin-like growth factor 2 binding proteins 2
IGF2BP3: insulin-like growth factor 2 binding proteins 3
SENP1: sentrin/sumo-specific proteases1
SENP3: sentrin/sumo-specific proteases3
